# Exploring cattle structural variation in the era of long reads, pangenome graphs, and near-complete assemblies

**DOI:** 10.1186/s40104-025-01294-7

**Published:** 2025-11-24

**Authors:** George E. Liu

**Affiliations:** Animal Genomics and Improvement Laboratory, Beltsville Agricultural Research Center, Agricultural Research Service, USDA, Beltsville, MD 20705 USA

**Keywords:** Cattle, Genome assembly, Genomic prediction, Long read sequencing, Pangenome graph, Structure variation

## Abstract

Structural variations (SVs ≥ 50 bp) are a critical but underexplored source of genetic diversity in cattle, shaping traits vital for productivity, adaptability, and health. Advances in long-read sequencing, pangenome graph construction, and near-complete genome assemblies now allow accurate SV detection and genotyping. These innovations overcome the limitations of single-reference genomes, enabling the discovery of complex SVs, including nested and overlapping variants, and providing access to previously inaccessible genomic regions such as centromeres and telomeres. This review highlights the current landscape of cattle SV research, with emphasis on integrating long-read sequencing and pangenome frameworks to uncover breed-specific and population-level variation. While many SVs are linked to economically important traits such as feed efficiency and disease resistance, their broader regulatory impacts remain an active area of investigation. Emerging functional genomics approaches, including transcriptomics, epigenomics, and genome editing, will clarify how SVs influence gene regulation and phenotype. Looking forward, the integration of SV catalogs with multi-omics data, imputation resources, and artificial intelligence-driven models will be essential for translating discoveries into breeding and conservation applications. Integrating structural variants into breeding pipelines promises to revolutionize livestock genomics, enabling precision selection and sustainable agriculture despite challenges in cost, data sharing, and functional validation.

## Introduction

**Fig. 1:**
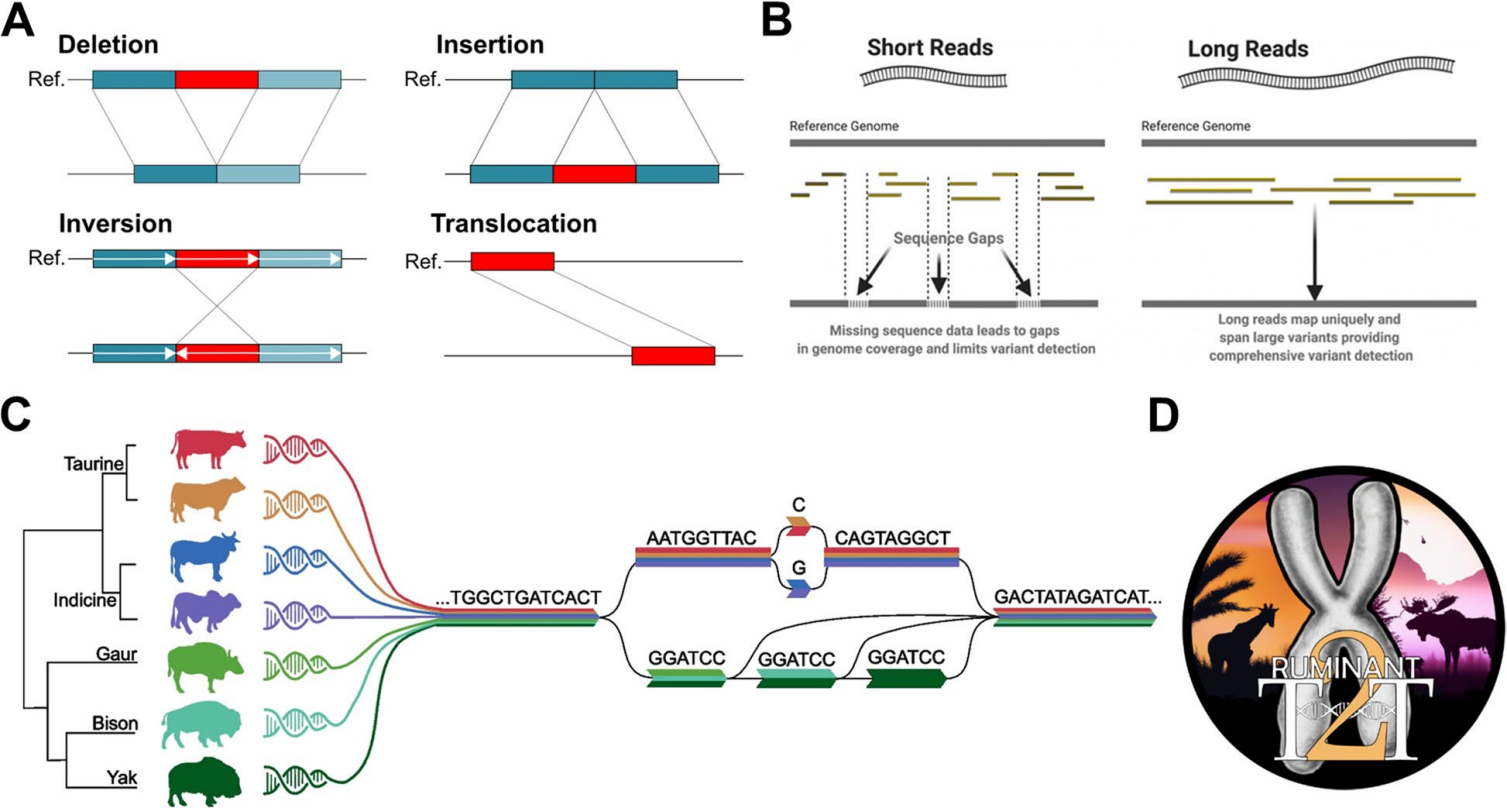
Investigating structural variation in cattle with long reads and pangenome graphs. **A** Types of structural variants (SVs) identified in cattle, including deletions, insertions, inversions, and translocations, highlighting the complexity of genomic variation. **B** Comparison of long-read versus short-read sequencing, illustrating the superior ability of long reads to detect and resolve SVs across the genome. **C** Conceptual representation of bovine species, subspecies, and breed relationships within a pangenome framework. Single-nucleotide variations and structural variations are modeled as alternative paths, capturing diversity across multiple lineages (modified from Smith et al. [13]). **D** Icon representing the Ruminant T2T Consortium, emphasizing the collaborative effort in generating near-complete ruminant genome assemblies

Livestock genomics has transformed agriculture by providing powerful tools to improve traits such as production efficiency, disease resistance, feed utilization, and adaptability. Among livestock, cattle are central to global food security and economic growth, with their genetic diversity shaped by domestication, selective breeding, and adaptation to diverse environments. Cattle, including *Bos taurus* and *Bos indicus*, exhibit extensive variation affecting milk production, meat quality, and resilience to disease. Unlocking the genetic basis of this diversity is essential for sustainable breeding programs, which require a full understanding of genetic variation. Genetic studies have made significant progress in identifying single-nucleotide polymorphisms (SNPs) associated with cattle production and health traits [1]. However, more complex forms of variation—structural variants (SVs ≥ 50 bp), including insertions, deletions, inversions, and translocations—cover larger genomic regions (Fig. 1A) and often exert stronger functional effects, such as altering gene dosage, modifying regulatory elements, or unmasking recessive alleles [2–5]. While SNP-based genome-wide association studies (GWAS) have identified thousands of variants linked to complex traits, they typically explain only a fraction of genetic variance, leaving much of the so-called “missing heritability” unresolved [6, 7]. Integrating SVs alongside SNPs offers a more complete understanding of the genomic architecture underlying complex traits [8]. In humans, SVs explain a substantial share of gene expression differences and are enriched at GWAS loci, particularly when larger than 20 kb [9–11]. Yet many SVs lie in repetitive regions, making them difficult to detect with short-read sequencing or SNP arrays [12].

Early cattle studies used microarrays and short-read sequencing to identify copy number variants and other SVs [14–21]. Our studies revealed that ~ 3.1% of the bovine genome comprises recent duplications (≥ 1 kb, ≥ 90% identity), often clustered in tandem arrays [22–24]. Genome-wide surveys across diverse breeds, supported by orthogonal validation methods like FISH and qPCR, linked SVs to parasite resistance, feed efficiency, and milk traits [25–27]. Roughly 75% of deletions and duplications were found in linkage disequilibrium with SNPs, but ~ 25% were not captured by SNP arrays, underscoring the need for SV-aware genotyping [19]. However, short-read sequencing approaches were limited, detecting only 30%–70% of SVs and often misclassifying variants in repetitive regions, with false discovery rates as high as 85%. Standard approaches such as read-pair, read-depth, and split-read analyses could not resolve repetitive or structurally complex regions—precisely where SVs are enriched. Moreover, most SV callers did not assign variants to haplotypes, restricting downstream association with complex traits.

Recognition of the limits of short-read sequencing in complex regions, as emphasized by the Genome in a Bottle Project [28–30], set the stage for long-read sequencing. Using Pacific Biosciences (PacBio) HiFi, Oxford Nanopore Technologies (ONT), and Hi-C technologies, the Telomere-to-Telomere (T2T) consortium has delivered the first human gapless genomes, resolving repetitive and structurally complex regions with unprecedented clarity [31–34]. The recent proliferation of high-quality, chromosome-level cattle assemblies mirrors trends in human genomics and provides new opportunities to close gaps and capture population diversity, opening the door to a deeper understanding of structural variation, adaptation, and trait biology. The pangenome concept, first introduced in microbial genomics, offers a powerful framework [35]. A pangenome captures both core sequences shared across individuals and variable sequences found in subsets of populations. Built from phased, haplotype-resolved assemblies, pangenomes improve SV discovery and support comparative genomics, evolutionary studies, and functional analyses such as epigenomics and metagenomics [10, 36–38]. While cattle CNVs and SVs have previously been reviewed using both short- and long-read data, this article focuses on cattle-specific advances while also synthesizing lessons from human genomics and placing cattle research within a broader comparative genomics framework that highlights recent pangenome and T2T developments.

## Long-read sequencing

The field of genome research has been transformed by long-read sequencing and complementary long-range mapping approaches, which together now deliver nearly complete genome assemblies and unprecedented SV resolution (Fig. 1B). PacBio HiFi sequencing, with error rates near 0.1% through circular consensus sequencing (CCS), has become a standard for variant discovery, assembly, and epigenomics [39, 40]. The recent PacBio Revio platform further expanded throughput (360 Gb/d), enabling sequencing of ~ 1,300 genomes per year at ~ $1,000 each, and incorporates DeepConsensus to improve accuracy [41]. ONT continues to complement this with ultra-long reads, often hundreds of kilobases in length, while Hi-C provides long-range chromatin contacts that aid phasing and scaffolding [42].

Two main strategies have emerged for SV detection using long reads: read-based and assembly-based approaches. Read-based methods map long reads to a reference genome using aligners such as Minimap2, NGMLR, or lra [43–45], followed by SV calling with programs like cuteSV [46], SVision [47], Sniffles2 [48], or pbsv. These approaches perform well at low coverage (~ 5 × HiFi) and handle heterozygous SVs and duplications, but are constrained by reference bias. In contrast, assembly-based approaches use de novo genome assemblies followed by whole-genome alignment with tools such as Hifiasm [49], SVIM-asm [50], and PAV [51]. These methods excel at discovering large insertions and novel sequences but require higher coverage (~ 20 ×) and more computational resources. The recent two benchmarking papers [52, 53] have outlined their advantages, limitations, input requirements, and examples of applications. They show that read-based methods achieve high recall at low coverage, while assembly-based methods provide broader variant classes and greater stability across datasets. Both reviews emphasized that integrating read- and assembly-based calls is essential for comprehensive SV discovery.

The same technologies are now being applied in livestock, enabling highly contiguous and accurate assemblies that improve SV detection and functional annotation. In cattle, such methods will advance the characterization of breed-specific variation, illuminate adaptive responses, and strengthen genomic prediction by capturing variants previously inaccessible with short-read sequencing. Together, long-read sequencing represents a major shift toward comprehensive catalogs of genomic diversity across livestock species.

## Pangenome graphs

The traditional reliance on single reference genomes, often derived from specific breeds, has introduced significant biases in livestock genomics, excluding breed-specific or rare SVs and limiting our understanding of within-species diversity. This limitation, together with the analytical challenges of high-resolution long-read data (PacBio, ONT), has driven a paradigm shift toward pangenomic approaches.

A pangenome captures the collective genomic diversity of a species, comprising both a core genome shared across all individuals and a variable genome present only in some (Fig. 1C). Unlike a single reference genome, which provides only a partial view, a pangenome provides a richer representation of structural, single-nucleotide, and insertion–deletion variation. When represented as a graph, shared sequences form nodes and alternative haplotypes form paths, with “bubbles” reflecting structural variants. Such frameworks improve both variant discovery and genotyping accuracy, particularly in repetitive and structurally complex regions.

The past few years have seen rapid progress in graph-based methodologies. Inspired by initiatives like the Human Pangenome Reference Consortium (HPRC), which aims to generate a diverse set of high-quality assemblies [34, 54], new computational frameworks have been developed, including vg (the Variation Graph toolkit) [55], Minigraph [56], Minigraph-Cactus (MC) [57], and the PanGenome Graph Builder (PGGB) [58]. These tools enable the construction of pangenome graphs with increasing sensitivity and scalability [59, 60]. Variation-aware genotyping tools such as PanGenie [51] and Giraffe [61] further extend these resources to short-read datasets, allowing efficient SV genotyping at a population scale [62]. They genotype SNPs and SVs against large graph references, achieving higher accuracy than linear mappers and enabling imputation panels derived from long-read-based catalogs. For long reads, emerging frameworks such as the Sequence Alignment Graph Algorithm (SAGA) [63] and graph-aware pipelines in Dynamic Read Analysis for GENomics (DRAGEN) [64] support SV calling, annotation, and genotyping within graph-based genomes, including the ability to augment graphs with novel alleles. These approaches, still maturing, promise to unify read-based and assembly-based methods in a graph framework that reduces reference bias and improves SV classification, especially in repetitive or complex genomic regions.

A recent graph-based human SV study used ONT to sequence 1,019 long-read genomes from 26 populations in the 1000 Genomes Project, identifying 167,291 sequence-resolved SVs and revealing mechanisms like LINE-1 and SVA transductions [63, 65]. It provides crucial insights into SV formation, especially involving repeat sequences and homology-mediated rearrangements, demonstrating the impact of long-read sequencing on understanding genomic architecture and aiding disease research. A companion paper reported a multi-ancestry SV imputation panel from 888 of the 1,019 samples [66]. They integrated their SVs with ~ 45 M variants from the 1000 Genomes Project Phase 3 and evaluated imputation accuracy using the UK Biobank. Metrics varied based on minor allele frequency, GIAB genomic region type (confident vs. difficult), and variant type, with simple insertions and deletions showing high imputation quality (mean concordance of 0.718 and 0.721; mean r_*2imp*_ = 0.921 and 0.924) in confident regions as compared to complex SVs in difficult regions. Although SVs had slightly lower imputation quality than SNVs, the difference was minimal. The SV reference panel provides a strong foundation for SV imputation and GWAS, identifying hundreds of independent SV associations and novel insights [66]. This demonstrates the value of incorporating SV analyses into workflows using the imputation panel.

Parallel progress is being made in cattle and other livestock. For example, the Pausch Lab used the Variation Graph toolkit and developed breed-specific and pan-genome reference graphs in cattle, showing their superior accuracy over traditional linear references, and uncovering 70 Mb of novel sequences [67–69]. Leonard et al. [70, 71] showed SV-based pangenomes from haplotype-resolved assemblies were highly consistent across platforms and algorithms, creating multi-species super-pangenomes with good consensus. They also constructed a pangenome from 16 PacBio HiFi cattle assemblies to identify SNPs and SVs [72]. After SV genotyping using short reads by PanGenie, researchers conducted molQTL mapping with testis transcriptome data, identifying 92 potential causal SV candidates. These studies collectively demonstrate the power of using variation-aware graph-based approaches in cattle genomics, providing a more accurate and comprehensive mapping of genetic variants compared to traditional linear references. These findings demonstrate the potential value of integrating pangenomic data into breeding programs, enhancing marker-assisted selection and genomic prediction models by accounting for SVs associated with desirable traits. Applications extend beyond trait discovery. By incorporating data from diverse breeds, cattle pangenomes reveal population-specific variation underlying environmental adaptation, such as heat tolerance in tropical breeds or cold resistance in temperate populations [73, 74]. Conservation also benefits: the Prendergast Lab integrated 116 Mb of novel African cattle sequences into reference assemblies, improving read mapping and SV detection, and helping preserve diversity in indigenous breeds [75]. Together, these advances show that graph-based pangenomes are transformative for cattle genomics, offering more complete and accurate variant catalogs than linear references.

## Advances in genome assemblies

Long-read sequencing has greatly advanced genome assembly quality, enabling highly accurate de novo assemblies across species. When combined with complementary methods such as Hi-C, which provides long-range scaffolding for detecting large SVs, these platforms deliver near-complete genomes with unprecedented contiguity and accuracy. Long reads bridge repetitive regions, allowing reconstruction of complex SVs such as tandem duplications and inversions that were previously unresolved. As a result, SV detection has markedly improved: high-quality assemblies support unbiased comparisons across individuals and breeds, capturing variants that short reads or single linear references often miss. Nearly complete assemblies now resolve centromeres, telomeres, and segmental duplications, uncovering SVs with important functional roles. Population-specific assemblies reveal adaptations such as disease resistance, while hybrid strategies combining HiFi, ONT, short reads, and Hi-C balance accuracy with cost-effectiveness. Haplotype phasing has advanced in parallel. Long reads and Hi-C enable phasing of both parental haplotypes across entire genomes, resolving heterozygous SNPs and SVs into contiguous haplotype blocks. Tools like HapCut2 [76], together with long, accurate HiFi reads, have increased the median length of phased blocks, while Hi-C extends them even further [33, 77]. The result is fully phased variation panels that improve detection of heterozygous SVs and enhance interpretation of complex traits, with direct applications to cattle breeding.

The landmark human T2T assemblies of CHM13 and HG0002 opened previously inaccessible genomic regions to SV discovery [32, 33, 78, 79]. In livestock, assemblies like goat ARS1 and cattle ARS-UCD2.0 achieved contig N50 sizes of ~ 20 Mb with near-complete fidelity [80, 81], setting benchmarks for animal genomics. Dozens of chromosome-level cattle assemblies have been released, including T2T or near-complete genomes for Holstein, sheep, and goat that filled reference gaps, especially in immunogenomic regions [82–85]. Pangenome efforts have expanded to sheep, *Bos indicus*, and yaks [86–88], reflecting a broader shift toward T2T and pangenomic frameworks. In cattle, three initiatives are spearheading progress:

1. Ruminant T2T (RT2T) Project – Led by Tim Smith, this project is generating complete diploid assemblies across ruminants, including cattle and sheep Y chromosomes, and nearly finished assemblies for multiple cattle breeds and relatives such as bison and river buffalo (Fig. 1D) [83, 89].
2. Bovine Pangenome Consortium (BPC) – Initiated by Ben Rosen, BPC is building a comprehensive bovine pangenome using ~ 15 breed-specific assemblies to improve SV and SNP detection at the genus level (Fig. 1C) [13].
3. Bovine Long Read Consortium (BLRC) – Led by Amanda Chamberlain, Ben Hayes, and colleagues, BLRC is extending the 1000 Bull Genomes Project into the long-read era to generate population-scale SV and SNP catalogs for genomic selection [4].

Similarly, in our recent pangenome study of 20 Holsteins and 10 Jerseys sequenced at 20 × HiFi coverage, we applied both read-based (cuteSV, SVision, Sniffles2, SVIM, pbsv) and assembly-based (SVIM-asm) approaches [90]. After filtering, we identified an average of ~ 28,500 high-confidence SVs per sample, predominantly insertions and deletions, with smaller numbers of duplications and inversions. This was a remarkable increase from short-read approaches, which usually detect 5,000 to 10,000 SVs per sample. Coverage experiments showed that 10 × HiFi achieves ~ 90% recall with a false positive rate of ~ 9%, balancing cost and accuracy. Cross-validation with orthogonal short-read SV calls supported ~ 74% of events [21]. Importantly, the inclusion of the Jersey genomes disproportionately increased the number of unique SVs, demonstrating the value of multi-breed sampling and the presence of breed-specific variation. These results highlight two key points: (1) population-scale SV catalogs require sequencing dozens of individuals per breed, not just a handful, to avoid missing substantial variation; and (2) long-read sequencing provides stable, high-confidence SV discovery, positioning cattle genomics to build resources comparable to those available in human genomics.

## Future perspectives and challenges

Recent advances in long-read sequencing, haplotype-resolved assemblies, and pangenome construction have fundamentally expanded our ability to characterize SV in cattle, moving beyond the limitations of short-read data. The Holstein- and Jersey-specific SV catalogs provide a strong foundation for exploring breed-specific variation [90]. Building on these resources, breed-specific phased pangenome graphs and large-scale SV imputation panels are poised to transform downstream applications, from more accurate variant genotyping to robust association studies across thousands of individuals. Looking ahead, integrating SV datasets into Artificial Intelligence (AI)-driven models could further improve predictive accuracy for complex traits, while cross-species pangenomes may uncover conserved and lineage-specific genetic variation important for adaptation and productivity. Coupled with functional genomics tools such as transcriptomics, epigenomics, and single-cell profiling, these strategies are expected to identify novel functional variants, refine trait mapping, and accelerate genomic selection, thereby enhancing genetic improvement and livestock management strategies.

Despite these advances, several challenges remain. Constructing and maintaining high-quality, breed-specific resources requires substantial sequencing and computational investment, which may restrict their broad adoption across diverse cattle populations. Unlike SNPs, which have vast public catalogs, SVs still lack comprehensive validated databases. Future studies must therefore develop shared SV resources to identify variant commonality across populations and to enable functional annotation. These resources will fill major knowledge gaps while providing direct applications in conservation and breeding, such as linking SVs to disease resistance, feed efficiency, or local adaptation. SV imputation and graph-based genotyping approaches, while powerful, must be further optimized to ensure accuracy across populations with varying ancestry and to integrate seamlessly with existing SNP-based genomic selection pipelines. Moreover, functional validation of SV–trait associations remains a bottleneck, demanding integration of multi-omics datasets, experimental models, and crossbreed comparisons. Overcoming these challenges will be essential for translating SV discoveries into practical breeding tools. Future efforts will also benefit from advances in AI approaches, as well as careful attention to ethical, regulatory, and data-sharing challenges.

## Conclusions

SVs are a critical driver of genetic diversity in cattle, influencing health, productivity, and adaptability. Advances in long-read sequencing, pangenome technology, and genome assemblies have revolutionized the study of SVs, enabling precise insights into genetic variations. These findings underscore the transformative potential of genomic research for improving cattle breeding and management strategies. Integrating SV studies into breeding programs and conservation efforts promises to address challenges like disease resistance and sustainability. Recent breakthroughs in sequencing and computational tools are bridging the gap between research and practical applications, paving the way for targeted genetic interventions. However, sustainable practices must guide these advancements to balance production goals with biodiversity conservation. The future of cattle genomics lies in comprehensive, collaborative, and innovative efforts. By harnessing multi-omics approaches, AI-driven analytics, and genome editing technologies, researchers can drive sustainable and resilient improvements in livestock populations, safeguarding genetic heritage and meeting the evolving needs of agriculture.

## Data Availability

Not applicable.

## Abbreviations

AI: Artificial Intelligence
BLRC: Bovine Long Read Consortium
BPC: Bovine Pangenome Consortium
CNV: Copy number variation
GIAB: Genome in a Bottle
GWAS: Genome-wide association studies
HPRC: Human Pangenome Reference Consortium
LD: Linkage disequilibrium
MC: Minigraph-Cactus
ONT: Oxford Nanopore Technologies
PGGB: PanGenome Graph Builder
RD: Read depth
RP: Read pair
SA: Sequence assembly
SMRT: Single Molecule Real Time
SNP: Single nucleotide polymorphism
SR: Split read
SV: Structural variation
T2T: Telomere-to-Telomere

## References

[1] Bovine HapMap Consortium, Gibbs RA, Taylor JF, Van Tassell CP, Barendse W, Eversole KA, et al. Genome-wide survey of SNP variation uncovers the genetic structure of cattle breeds. Science. 2009;324(5926):528–32. 10.1126/science.1167936. 19390050 10.1126/science.1167936 PMC2735092

[2] Scherer SW, Lee C, Birney E, Altshuler DM, Eichler EE, Carter NP, et al. Challenges and standards in integrating surveys of structural variation. Nat Genet. 2007;39(7 Suppl):S7–15. 10.1038/ng2093. 17597783 10.1038/ng2093 PMC2698291

[3] Bickhart DM, Liu GE. The challenges and importance of structural variation detection in livestock. Front Genet. 2014;5:37. 10.3389/fgene.2014.00037. 24600474 10.3389/fgene.2014.00037 PMC3927395

[4] Nguyen TV, Vander Jagt CJ, Wang JH, Daetwyler HD, Xiang RD, Goddard ME, et al. In it for the long run: perspectives on exploiting long-read sequencing in livestock for population scale studies of structural variants. Genet Sel Evol. 2023;55:25. 10.1186/s12711-023-00800-7. 36721111 10.1186/s12711-023-00783-5 PMC9887926

[5] Zhang F, Gu WL, Hurles ME, Lupski JR. Copy number variation in human health, disease, and evolution. Annu Rev Genomics Hum Genet. 2009;10:451–81. 10.1146/annurev.genom.9.081307.164217. 19715442 10.1146/annurev.genom.9.081307.164217 PMC4472309

[6] Marouli E, Graff M, Medina-Gomez C, Lo KS, Wood AR, Kjaer TR, et al. Rare and low-frequency coding variants alter human adult height. Nature. 2017;542(7640):186–90. 10.1038/nature21039. 28146470 10.1038/nature21039 PMC5302847

[7] Yengo L, Vedantam S, Marouli E, Sidorenko J, Bartell E, Sakaue S, et al. A saturated map of common genetic variants associated with human height. Nature. 2022;610(7933):704–12. 10.1038/s41586-022-05275-y. 36224396 10.1038/s41586-022-05275-y PMC9605867

[8] Manolio TA, Collins FS, Cox NJ, Goldstein DB, Hindorff LA, Hunter DJ, et al. Finding the missing heritability of complex diseases. Nature. 2009;461(7265):747–53. 10.1038/nature08494. 19812666 10.1038/nature08494 PMC2831613

[9] Stranger BE, Forrest MS, Dunning M, Ingle CE, Beazley C, Thorne N, et al. Relative impact of nucleotide and copy number variation on gene expression phenotypes. Science. 2007;315(5813):848–53. 10.1126/science.1136678. 17289997 10.1126/science.1136678 PMC2665772

[10] Chaisson MJP, Huddleston J, Dennis MY, Sudmant PH, Malig M, Hormozdiari F, et al. Resolving the complexity of the human genome using single-molecule sequencing. Nature. 2015;517(7536):608–11. 10.1038/nature13907. 25383537 10.1038/nature13907 PMC4317254

[11] Sudmant PH, Rausch T, Gardner EJ, Handsaker RE, Abyzov A, Huddleston J, et al. An integrated map of structural variation in 2,504 human genomes. Nature. 2015;526(7571):75–81. 10.1038/nature15394. 26432246 10.1038/nature15394 PMC4617611

[12] Estivill X, Armengol L. Copy number variants and common disorders: filling the gaps and exploring complexity in genome-wide association studies. PLoS Genet. 2007;3(10):e190. 10.1371/journal.pgen.0030190. 17953491 10.1371/journal.pgen.0030190 PMC2039766

[13] Smith TPL, Bickhart DM, Boichard D, Chamberlain AJ, Djikeng A, Jiang Y, et al. The bovine pangenome consortium: democratizing production and accessibility of genome assemblies for global cattle breeds and other bovine species. Genome Biol. 2023;24:139. 10.1186/s13059-023-02975-0. 37337218 10.1186/s13059-023-02975-0 PMC10278262

[14] Fadista J, Thomsen B, Holm LE, Bendixen C. Copy number variation in the bovine genome. BMC Genomics. 2010;11:284. 10.1186/1471-2164-11-284. 20459598 10.1186/1471-2164-11-284 PMC2902221

[15] Bae JS, Cheong HS, Kim LH, NamGung S, Park TJ, Chun JY, et al. Identification of copy number variations and common deletion polymorphisms in cattle. BMC Genomics. 2010;11:232. 10.1186/1471-2164-11-232. 20377913 10.1186/1471-2164-11-232 PMC2859865

[16] Cicconardi F, Chillemi G, Tramontano A, Marchitelli C, Valentini A, Ajmone-Marsan P, et al. Massive screening of copy number population-scale variation in *Bos taurus* genome. BMC Genomics. 2013;14:124. 10.1186/1471-2164-14-124. 23442185 10.1186/1471-2164-14-124 PMC3618309

[17] Keel BN, Keele JW, Snelling WM. Genome-wide copy number variation in the bovine genome detected using low coverage sequence of popular beef breeds. Anim Genet. 2017;48(2):141–50. 10.1111/age.12519. 27775157 10.1111/age.12519

[18] Bickhart DM, Xu LY, Hutchison JL, Cole JB, Null DJ, Schroeder SG, et al. Diversity and population-genetic properties of copy number variations and multicopy genes in cattle. DNA Res. 2016;23(3):253–62. 10.1093/dnares/dsw013. 27085184 10.1093/dnares/dsw013 PMC4909312

[19] Xu LY, Cole JB, Bickhart DM, Hou YL, Song JZ, VanRaden PM, et al. Genome wide CNV analysis reveals additional variants associated with milk production traits in Holsteins. BMC Genomics. 2014;15:683. 10.1186/1471-2164-15-683. 25128478 10.1186/1471-2164-15-683 PMC4152564

[20] Zhou Y, Utsunomiya YT, Xu LY, Hay EHA, Bickhart DM, Almeida Alexandre P, et al. Genome-wide CNV analysis reveals variants associated with growth traits in *Bos indicus*. BMC Genomics. 2016;17:419. 10.1186/s12864-016-2461-4. 27245577 10.1186/s12864-016-2461-4 PMC4888316

[21] Zhou Y, Yang L, Han XT, Han JZ, Hu Y, Li F, et al. Assembly of a pangenome for global cattle reveals missing sequences and novel structural variations, providing new insights into their diversity and evolutionary history. Genome Res. 2022;32(8):1585–601. 10.1101/gr.276550.122. 35977842 10.1101/gr.276550.122 PMC9435747

[22] Liu GE, Ventura M, Cellamare A, Chen L, Cheng Z, Zhu B, et al. Analysis of recent segmental duplications in the bovine genome. BMC Genomics. 2009;10:571. 10.1186/1471-2164-10-571. 19951423 10.1186/1471-2164-10-571 PMC2796684

[23] Bickhart DM, Hou YL, Schroeder SG, Alkan C, Cardone MF, Matukumalli LK, et al. Copy number variation of individual cattle genomes using next-generation sequencing. Genome Res. 2012;22(4):778–90. 10.1101/gr.133967.111. 22300768 10.1101/gr.133967.111 PMC3317159

[24] Liu GE, Hou YL, Zhu B, Cardone MF, Jiang L, Cellamare A, et al. Analysis of copy number variations among diverse cattle breeds. Genome Res. 2010;20(5):693–703. 10.1101/gr.105403.110. 20212021 10.1101/gr.105403.110 PMC2860171

[25] Hou YL, Liu GE, Bickhart DM, Matukumalli LK, Li CJ, Song JZ, et al. Genomic regions showing copy number variations associate with resistance or susceptibility to gastrointestinal nematodes in Angus cattle. Funct Integr Genomics. 2012;12(1):81–92. 10.1007/s10142-011-0252-1. 21928070 10.1007/s10142-011-0252-1

[26] Zhou Y, Connor EE, Wiggans GR, Lu YF, Tempelman RJ, Schroeder SG, et al. Genome-wide copy number variant analysis reveals variants associated with 10 diverse production traits in Holstein cattle. BMC Genomics. 2018;19:314. 10.1186/s12864-018-4699-5. 29716533 10.1186/s12864-018-4699-5 PMC5930521

[27] Hay EHA, Utsunomiya YT, Xu LY, Zhou Y, Neves HHR, Carvalheiro R, et al. Genomic predictions combining SNP markers and copy number variations in Nellore cattle. BMC Genomics. 2018;19:441. 10.1186/s12864-018-4787-6. 29871610 10.1186/s12864-018-4787-6 PMC5989480

[28] Zook JM, Catoe D, McDaniel J, Vang L, Spies N, Sidow A, et al. Extensive sequencing of seven human genomes to characterize benchmark reference materials. Sci Data. 2016;3:160025. 10.1038/sdata.2016.25. 27271295 10.1038/sdata.2016.25 PMC4896128

[29] Zook JM, McDaniel J, Olson ND, Wagner J, Parikh H, Heaton H, et al. An open resource for accurately benchmarking small variant and reference calls. Nat Biotechnol. 2019;37(5):561–6. 10.1038/s41587-019-0074-6. 30936564 10.1038/s41587-019-0074-6 PMC6500473

[30] Dwarshuis N, Kalra D, McDaniel J, Sanio P, Alvarez Jerez P, Jadhav B, et al. The GIAB genomic stratifications resource for human reference genomes. Nat Commun. 2024;15:9029. 10.1038/s41467-024-53260-y. 39424793 10.1038/s41467-024-53260-y PMC11489684

[31] Rhie A, Nurk S, Cechova M, Hoyt SJ, Taylor DJ, Altemose N, et al. The complete sequence of a human Y chromosome. Nature. 2023;621(7978):344–54. 10.1038/s41586-023-06457-y. 37612512 10.1038/s41586-023-06457-y PMC10752217

[32] Nurk S, Koren S, Rhie A, Rautiainen M, Bzikadze AV, Mikheenko A, et al. The complete sequence of a human genome. Science. 2022;376(6588):44–53. 10.1126/science.abj6987. 35357919 10.1126/science.abj6987 PMC9186530

[33] Logsdon GA, Vollger MR, Hsieh P, Mao YF, Liskovykh MA, Koren S, et al. The structure, function and evolution of a complete human chromosome 8. Nature. 2021;593(7857):101–7. 10.1038/s41586-021-03420-7. 33828295 10.1038/s41586-021-03420-7 PMC8099727

[34] Miga KH, Wang T. The need for a human pangenome reference sequence. Annu Rev Genom Hum Genet. 2021;22(1):81–102. 10.1146/annurev-genom-120120-081921. 10.1146/annurev-genom-120120-081921 PMC8410644 33929893

[35] Tettelin H, Masignani V, Cieslewicz MJ, Donati C, Medini D, Ward NL, et al. Genome analysis of multiple pathogenic isolates of *Streptococcus agalactiae*: implications for the microbial “pan-genome". Proc Natl Acad Sci U S A. 2005;102(39):13950–5. 10.1073/pnas.0506758102. 16172379 10.1073/pnas.0506758102 PMC1216834

[36] Kidd JM, Cooper GM, Donahue WF, Hayden HS, Sampas N, Graves T, et al. Mapping and sequencing of structural variation from eight human genomes. Nature. 2008;453(7191):56–64. 10.1038/nature06862. 18451855 10.1038/nature06862 PMC2424287

[37] Wheeler DA, Srinivasan M, Egholm M, Shen YF, Chen L, McGuire A, et al. The complete genome of an individual by massively parallel DNA sequencing. Nature. 2008;452(7189):872–6. 10.1038/nature06884. 18421352 10.1038/nature06884

[38] Huddleston J, Chaisson MJP, Steinberg KM, Warren W, Hoekzema K, Gordon D, et al. Discovery and genotyping of structural variation from long-read haploid genome sequence data. Genome Res. 2017;27(5):677–85. 10.1101/gr.214007.116. 27895111 10.1101/gr.214007.116 PMC5411763

[39] Ni P, Nie F, Zhong ZY, Xu JR, Huang N, Zhang J, et al. DNA 5-methylcytosine detection and methylation phasing using PacBio circular consensus sequencing. Nat Commun. 2023;14:4054. 10.1038/s41467-023-39784-9. 37422489 10.1038/s41467-023-39784-9 PMC10329642

[40] Olivia Tse OY, Jiang PY, Cheng SH, Peng WL, Shang HM, Wong J, et al. Genome-wide detection of cytosine methylation by single molecule real-time sequencing. Proc Natl Acad Sci U S A. 2021;118(5):e2019768118. 10.1073/pnas.2019768118. 33495335 10.1073/pnas.2019768118 PMC7865158

[41] Baid G, Cook DE, Shafin K, Yun T, Llinares-López F, Berthet Q, et al. DeepConsensus improves the accuracy of sequences with a gap-aware sequence transformer. Nat Biotechnol. 2023;41(2):232–8. 10.1038/s41587-022-01435-7. 36050551 10.1038/s41587-022-01435-7

[42] Lieberman-Aiden E, van Berkum NL, Williams L, Imakaev M, Ragoczy T, Telling A, et al. Comprehensive mapping of long-range interactions reveals folding principles of the human genome. Science. 2009;326(5950):289–93. 10.1126/science.1181369. 19815776 10.1126/science.1181369 PMC2858594

[43] Li H. Minimap2: pairwise alignment for nucleotide sequences. Bioinformatics. 2018;34(18):3094–100. 10.1093/bioinformatics/bty191. 29750242 10.1093/bioinformatics/bty191 PMC6137996

[44] Sedlazeck FJ, Rescheneder P, Smolka M, Fang H, Nattestad M, von Haeseler A, et al. Accurate detection of complex structural variations using single-molecule sequencing. Nat Methods. 2018;15(6):461–8. 10.1038/s41592-018-0001-7. 29713083 10.1038/s41592-018-0001-7 PMC5990442

[45] Ren JW, Chaisson MJP. Lra: a long read aligner for sequences and contigs. PLoS Comput Biol. 2021;17(6):e1009078. 10.1371/journal.pcbi.1009078. 34153026 10.1371/journal.pcbi.1009078 PMC8248648

[46] Jiang T, Liu YZ, Jiang Y, Li JY, Gao Y, Cui Z, et al. Long-read-based human genomic structural variation detection with cuteSV. Genome Biol. 2020;21:189. 10.1186/s13059-020-02107-y. 32746918 10.1186/s13059-020-02107-y PMC7477834

[47] Lin JD, Wang SB, Audano PA, Meng DY, Flores JI, Kosters W, et al. Svision: a deep learning approach to resolve complex structural variants. Nat Methods. 2022;19(10):1230–3. 10.1038/s41592-022-01609-w. 36109679 10.1038/s41592-022-01609-w PMC9985066

[48] Smolka M, Paulin LF, Grochowski CM, Horner DW, Mahmoud M, Behera S, et al. Detection of mosaic and population-level structural variants with Sniffles2. Nat Biotechnol. 2024;42(10):1571–80. 10.1038/s41587-023-02024-y. 38168980 10.1038/s41587-023-02024-y PMC11217151

[49] Cheng HY, Concepcion GT, Feng XW, Zhang HW, Li H. Haplotype-resolved *de novo* assembly using phased assembly graphs with hifiasm. Nat Methods. 2021;18(2):170–5. 10.1038/s41592-020-01056-5. 33526886 10.1038/s41592-020-01056-5 PMC7961889

[50] Heller D, Vingron M. Svim-asm: structural variant detection from haploid and diploid genome assemblies. Bioinformatics. 2021;36(22–23):5519–21. 10.1093/bioinformatics/btaa1034. 33346817 10.1093/bioinformatics/btaa1034 PMC8016491

[51] Ebert P, Audano PA, Zhu QH, Rodriguez-Martin B, Porubsky D, Bonder MJ, et al. Haplotype-resolved diverse human genomes and integrated analysis of structural variation. Science. 2021;372(6537):eabf7117. 10.1126/science.abf7117. 33632895 10.1126/science.abf7117 PMC8026704

[52] Lin JD, Jia P, Wang SB, Kosters W, Ye K. Comparison and benchmark of structural variants detected from long read and long-read assembly. Brief Bioinform. 2023;24(4):bbad188. 10.1093/bib/bbad188. 37200087 10.1093/bib/bbad188

[53] Liu YH, Luo C, Golding SG, Ioffe JB, Zhou XM. Tradeoffs in alignment and assembly-based methods for structural variant detection with long-read sequencing data. Nat Commun. 2024;15:2447. 10.1038/s41467-024-46614-z. 38503752 10.1038/s41467-024-46614-z PMC10951360

[54] Wang T, Antonacci-Fulton L, Howe K, Lawson HA, Lucas JK, Phillippy AM, et al. The human pangenome project: a global resource to map genomic diversity. Nature. 2022;604(7906):437–46. 10.1038/s41586-022-04601-8. 35444317 10.1038/s41586-022-04601-8 PMC9402379

[55] Hickey G, Heller D, Monlong J, Sibbesen JA, Sirén J, Eizenga J, et al. Genotyping structural variants in pangenome graphs using the vg toolkit. Genome Biol. 2020;21:35. 10.1186/s13059-020-1941-7. 32051000 10.1186/s13059-020-1941-7 PMC7017486

[56] Li H, Feng XW, Chu C. The design and construction of reference pangenome graphs with minigraph. Genome Biol. 2020;21:265. 10.1186/s13059-020-02168-z. 33066802 10.1186/s13059-020-02168-z PMC7568353

[57] Hickey G, Monlong J, Ebler J, Novak AM, Eizenga JM, Gao Y, et al. Pangenome graph construction from genome alignments with Minigraph-Cactus. Nat Biotechnol. 2024;42:663–73. 10.1038/s41587-023-01793-w. 37165083 10.1038/s41587-023-01793-w PMC10638906

[58] Garrison E, Guarracino A, Heumos S, Villani F, Bao ZG, Tattini L, et al. Building pangenome graphs. Nat Methods. 2024;21(11):2008–12. 10.1038/s41592-024-02430-3. 39433878 10.1038/s41592-024-02430-3

[59] Andreace F, Lechat P, Dufresne Y, Chikhi R. Comparing methods for constructing and representing human pangenome graphs. Genome Biol. 2023;24:274. 10.1186/s13059-023-03098-2. 38037131 10.1186/s13059-023-03098-2 PMC10691155

[60] Liao WW, Asri M, Ebler J, Doerr D, Haukness M, Hickey G, et al. A draft human pangenome reference. Nature. 2023;617(7960):312–24. 10.1038/s41586-023-05896-x. 37165242 10.1038/s41586-023-05896-x PMC10172123

[61] Sirén J, Monlong J, Chang X, Novak AM, Eizenga JM, Markello C, et al. Pangenomics enables genotyping of known structural variants in 5202 diverse genomes. Science. 2021;374(6574):abg8871. 10.1126/science.abg8871. 34914532 10.1126/science.abg8871 PMC9365333

[62] Du ZZ, He JB, Jiao WB. A comprehensive benchmark of graph-based genetic variant genotyping algorithms on plant genomes for creating an accurate ensemble pipeline. Genome Biol. 2024;25:91. 10.1186/s13059-024-03239-1. 38589937 10.1186/s13059-024-03239-1 PMC11003132

[63] Schloissnig S, Pani S, Ebler J, Hain C, Tsapalou V, Söylev A, et al. Structural variation in 1019 diverse humans based on long-read sequencing. Nature. 2025;644(8076):442–52. 10.1038/s41586-025-09290-7. 40702182 10.1038/s41586-025-09290-7 PMC12350158

[64] Behera S, Catreux S, Rossi M, Truong S, Huang Z, Ruehle M, et al. Comprehensive and accurate genome analysis at scale using DRAGEN accelerated algorithms. bioRxiv [Preprint]. 2024. 10.1101/2024.01.02.573821.

[65] Schloissnig S, Pani S, Rodriguez-Martin B, Ebler J, Hain C, Tsapalou V, et al. Structural variation in 1,019 diverse humans based on long-read sequencing. Nature. 2025;644:442–52. 10.1038/s41586-025-09290-7. 40702182 10.1038/s41586-025-09290-7 PMC12350158

[66] Noyvert B, Erzurumluoglu AM, Drichel D, Omland S, Andlauer TFM, Mueller S, et al. Imputation of structural variants using a multi-ancestry long-read sequencing panel enables identification of disease associations. eLife. 2025;14:RP106115. 10.7554/eLife.106115.1.

[67] Crysnanto D, Wurmser C, Pausch H. Accurate sequence variant genotyping in cattle using variation-aware genome graphs. Genet Sel Evol. 2019;51:21. 10.1186/s12711-019-0462-x. 31092189 10.1186/s12711-019-0462-x PMC6521551

[68] Crysnanto D, Pausch H. Bovine breed-specific augmented reference graphs facilitate accurate sequence read mapping and unbiased variant discovery. Genome Biol. 2020;21:184. 10.1186/s13059-020-02105-0. 32718320 10.1186/s13059-020-02105-0 PMC7385871

[69] Crysnanto D, Leonard AS, Fang ZH, Pausch H. Novel functional sequences uncovered through a bovine multiassembly graph. Proc Natl Acad Sci U S A. 2021;118(20):e2101056118. 10.1073/pnas.2101056118. 33972446 10.1073/pnas.2101056118 PMC8157972

[70] Leonard AS, Crysnanto D, Fang ZH, Heaton MP, Vander Ley BL, Herrera C, et al. Structural variant-based pangenome construction has low sensitivity to variability of haplotype-resolved bovine assemblies. Nat Commun. 2022;13:3012. 10.1038/s41467-022-30680-2. 35641504 10.1038/s41467-022-30680-2 PMC9156671

[71] Leonard AS, Crysnanto D, Mapel XM, Bhati M, Pausch H. Graph construction method impacts variation representation and analyses in a bovine super-pangenome. Genome Biol. 2023;24:124. 10.1186/s13059-023-02969-y. 37217946 10.1186/s13059-023-02969-y PMC10204317

[72] Leonard AS, Mapel XM, Pausch H. Pangenome-genotyped structural variation improves molecular phenotype mapping in cattle. Genome Res. 2024;34(2):300–9. 10.1101/gr.278267.123. 38355307 10.1101/gr.278267.123 PMC10984387

[73] Cooke RF, Daigle CL, Moriel P, Smith SB, Tedeschi LO, Vendramini JMB. Cattle adapted to tropical and subtropical environments: social, nutritional, and carcass quality considerations. J Anim Sci. 2020;98(2):skaa014. 10.1093/jas/skaa014. 31955200 10.1093/jas/skaa014 PMC7023624

[74] Cooke RF, Cardoso RC, Cerri RLA, Lamb GC, Pohler KG, Riley DG, et al. Cattle adapted to tropical and subtropical environments: genetic and reproductive considerations. J Anim Sci. 2020;98(2):skaa015. 10.1093/jas/skaa015. 31955201 10.1093/jas/skaa015 PMC7032034

[75] Talenti A, Powell J, Hemmink JD, Cook EAJ, Wragg D, Jayaraman S, et al. A cattle graph genome incorporating global breed diversity. Nat Commun. 2022;13:910. 10.1038/s41467-022-28605-0. 35177600 10.1038/s41467-022-28605-0 PMC8854726

[76] Edge P, Bafna V, Bansal V. HapCUT2: robust and accurate haplotype assembly for diverse sequencing technologies. Genome Res. 2017;27(5):801–12. 10.1101/gr.213462.116. 27940952 10.1101/gr.213462.116 PMC5411775

[77] Makova KD, Pickett BD, Harris RS, Hartley GA, Cechova M, Pal K, et al. The complete sequence and comparative analysis of ape sex chromosomes. Nature. 2024;630(8016):401–11. 10.1038/s41586-024-07473-2. 38811727 10.1038/s41586-024-07473-2 PMC11168930

[78] Miga KH, Koren S, Rhie A, Vollger MR, Gershman A, Bzikadze A, et al. Telomere-to-telomere assembly of a complete human X chromosome. Nature. 2020;585(7823):79–84. 10.1038/s41586-020-2547-7. 32663838 10.1038/s41586-020-2547-7 PMC7484160

[79] Jarvis ED, Formenti G, Rhie A, Guarracino A, Yang CT, Wood J, et al. Semi-automated assembly of high-quality diploid human reference genomes. Nature. 2022;611(7936):519–31. 10.1038/s41586-022-05325-5. 36261518 10.1038/s41586-022-05325-5 PMC9668749

[80] Bickhart DM, Rosen BD, Koren S, Sayre BL, Hastie AR, Chan S, et al. Single-molecule sequencing and chromatin conformation capture enable *de novo* reference assembly of the domestic goat genome. Nat Genet. 2017;49(4):643–50. 10.1038/ng.3802. 28263316 10.1038/ng.3802 PMC5909822

[81] Rosen BD, Bickhart DM, Schnabel RD, Koren S, Elsik CG, Tseng E, et al. *De novo* assembly of the cattle reference genome with single-molecule sequencing. Gigascience. 2020;9(3):giaa021. 10.1093/gigascience/giaa021. 32191811 10.1093/gigascience/giaa021 PMC7081964

[82] Li TT, Xia T, Wu JQ, Hong H, Sun ZL, Wang M, et al. *De novo* genome assembly depicts the immune genomic characteristics of cattle. Nat Commun. 2023;14:6601. 10.1038/s41467-023-42161-1. 37857610 10.1038/s41467-023-42161-1 PMC10587341

[83] Olagunju TA, Rosen BD, Neibergs HL, Becker GM, Davenport KM, Elsik CG, et al. Telomere-to-telomere assemblies of cattle and sheep Y-chromosomes uncover divergent structure and gene content. Nat Commun. 2024;15:8277. 10.1038/s41467-024-52384-5. 39333471 10.1038/s41467-024-52384-5 PMC11436988

[84] Wu H, Luo LY, Zhang YH, Zhang CY, Huang JH, Mo DX, et al. Telomere-to-telomere genome assembly of a male goat reveals variants associated with Cashmere traits. Nat Commun. 2024;15:10041. 10.1038/s41467-024-54188-z. 39567477 10.1038/s41467-024-54188-z PMC11579321

[85] Luo LY, Wu H, Zhao LM, Zhang YH, Huang JH, Liu QY, et al. Telomere-to-telomere sheep genome assembly identifies variants associated with wool fineness. Nat Genet. 2025;57(1):218–30. 10.1038/s41588-024-02037-6. 39779954 10.1038/s41588-024-02037-6

[86] Dai XL, Bian PP, Hu DX, Luo FN, Huang YZ, Jiao SH, et al. A Chinese indicine pangenome reveals a wealth of novel structural variants introgressed from other *Bos* species. Genome Res. 2023;33(8):1284–98. 10.1101/gr.277481.122. 37714713 10.1101/gr.277481.122 PMC10547261

[87] Li R, Gong M, Zhang XM, Wang F, Liu ZY, Zhang L, et al. A sheep pangenome reveals the spectrum of structural variations and their effects on tail phenotypes. Genome Res. 2023;33(3):463–77. 10.1101/gr.277372.122. 37310928 10.1101/gr.277372.12 PMC10078295

[88] Lan DL, Fu W, Ji WH, Mipam TD, Xiong XR, Ying S, et al. Pangenome and multi-tissue gene atlas provide new insights into the domestication and highland adaptation of yaks. J Anim Sci Biotechnol. 2024;15:64. 10.1186/s40104-024-01027-2. 38706000 10.1186/s40104-024-01027-2 PMC11071219

[89] Kalbfleisch TS, McKay SD, Murdoch BM, Adelson DL, Almansa-Villa D, Becker G, et al. The ruminant telomere-to-telomere (RT2T) consortium. Nat Genet. 2024;56(8):1566–73. 10.1038/s41588-024-01835-2. 39103649 10.1038/s41588-024-01835-2

[90] Gao YH, Yang L, Kuhn K, Li WL, Zanton G, Bowman M, et al. Long read and preliminary pangenome analyses reveal breed-specific structural variations and novel sequences in Holstein and Jersey cattle. J Adv Res. 2025:S2090–S1232(25)00258–9. 10.1016/j.jare.2025.04.014.10.1016/j.jare.2025.04.01440258473

